# Recent advancements and prospects in noble and non-noble electrocatalysts for materials methanol oxidation reactions

**DOI:** 10.1186/s11671-024-04066-w

**Published:** 2024-08-14

**Authors:** Monika Singh, Hari Mohan Sharma, Ram K. Gupta, Anuj Kumar

**Affiliations:** 1 Department of Chemistry, GLA University, Mathura-281406, India; 2 Department of Chemistry, Pittsburg State University, Pittsburg, KS 66762 USA; 3 National Institute of Material Advancement, Pittsburg, KS 66762 USA

**Keywords:** Electrocatalytic methanol oxidation reaction, Noble and non-noble catalysts, Single-atom catalysts, Molecular catalysts

## Abstract

The direct methanol fuel cell (DMFC) represents a highly promising alternative power source for small electronics and automobiles due to its low operating temperatures, high efficiency, and energy density. The methanol oxidation process (MOR) constitutes a fundamental chemical reaction occurring at the positive electrode of a DMFC. Pt-based materials serve as widely utilized MOR electrocatalysts in DMFCs. Nevertheless, various challenges, such as sluggish reaction rates, high production costs primarily attributed to the expensive Pt-based catalyst, and the adverse effects of CO poisoning on the Pt catalysts, hinder the commercialization of DMFCs. Consequently, endeavors to identify an alternative catalyst to Pt-based catalysts that mitigate these drawbacks represent a critical focal point of DMFC research. In pursuit of this objective, researchers have developed diverse classes of MOR electrocatalysts, encompassing those derived from noble and non-noble metals. This review paper delves into the fundamental concept of MOR and its operational mechanisms, as well as the latest advancements in electrocatalysts derived from noble and non-noble metals, such as single-atom and molecule catalysts. Moreover, a comprehensive analysis of the constraints and prospects of MOR electrocatalysts, encompassing those based on noble metals and those based on non-noble metals, has been undertaken.

## Introduction

**Fig. 1:**
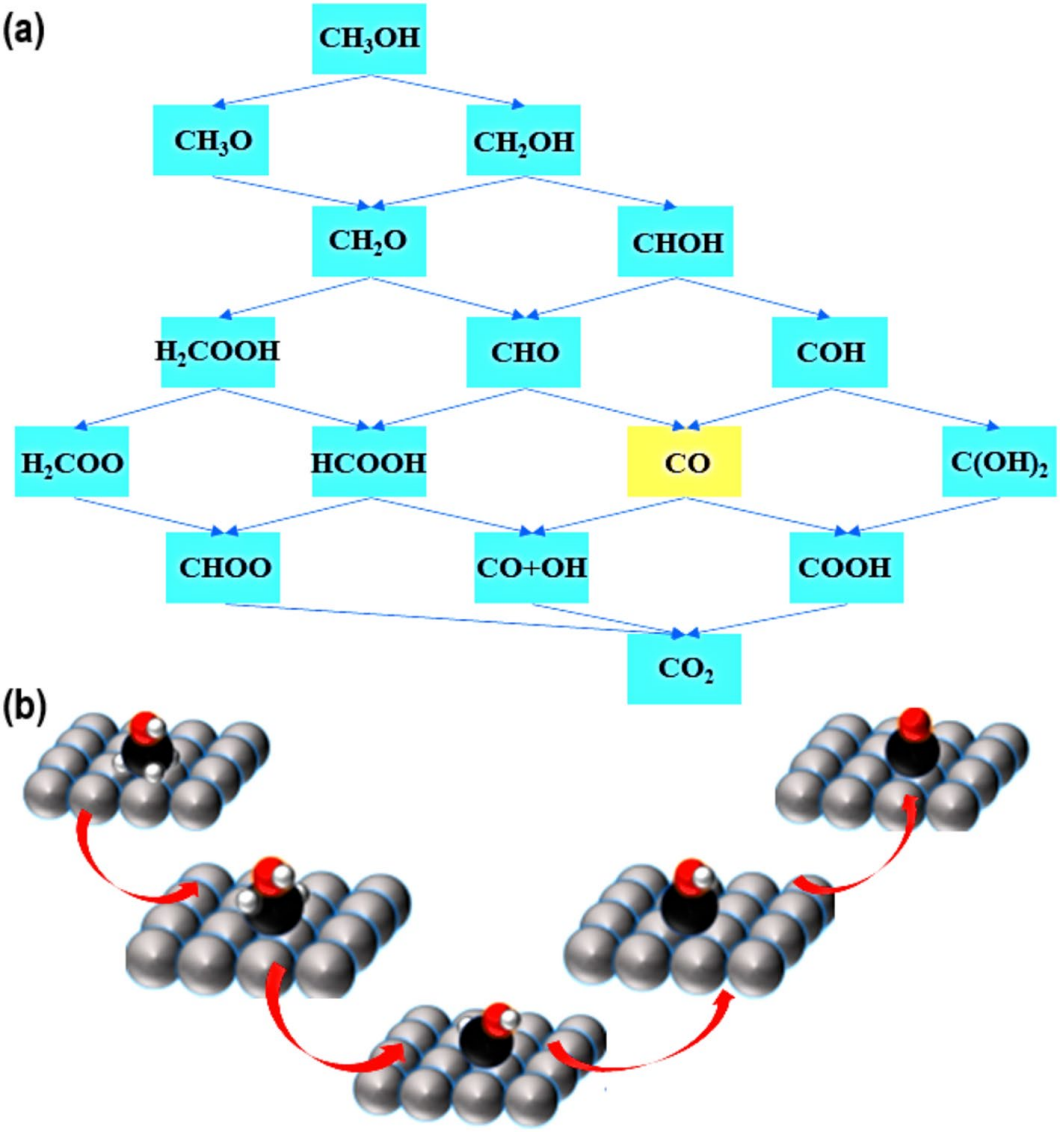
**a** The diagram illustrates a typical CH_3_OH oxidation reaction, showcasing various reaction intermediates and products. **b** The diagram illustrates the progressive removal of hydrogen atoms throughout the CH_3_OH oxidation process. Adapted with permission [1]. Copyright © 2022, Taylor & Francis

The methanol oxidation reaction (MOR) typically involves complex chemical events that indirectly produce CO_2_ [1]. This process includes the generation of intermediates through several reaction pathways, as depicted in Fig. 1a, b [2]. As one of the intermediates in the MOR process, CO slows down the reaction and makes it less effective by blocking the electrocatalysts' catalytic active sites [3]. Scientists are searching for efficient electrocatalysts that contain either a single metal or platinum combined with other transition metals (TMs) to enhance catalytic performance and prevent CO poisoning [4]. This occurs because CO can be expelled from the catalyst's active sites by facilitating its oxidation with H_2_O or OH^‒^ ions [5, 6]. Although this technique effectively reduces the amount of Pt used in the catalyst ink, investigators are currently trying to substitute Pt-group metals (PGMs) with non-noble metal electrocatalysts for alcohol oxidation processes [7].

**Fig. 2:**
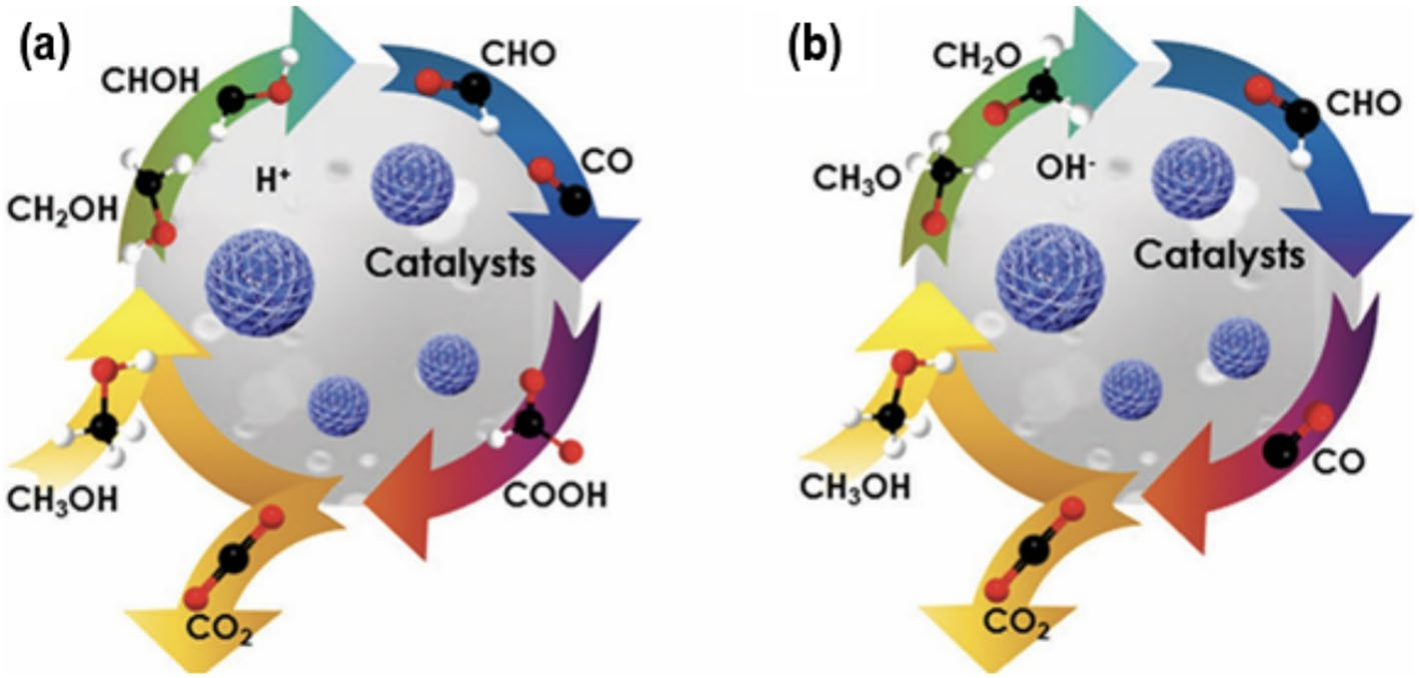
Depiction of MOR mechanism on the catalyst's surface under **a** acidic and **b** basic conditions. Adapted with permission [16]. Copyright © 2019, WILEY–VCH

It is desirable to assess the reaction mechanism and kinetics of MOR in acidic as well as alkaline mediums along with essential requirements of electrocatalysts before focusing on the effect of carbon-based support on electrocatalysts for MOR of fuel cells. MOR occurs either in acidic or basic environments, however, alkaline electrolyte supports the development of inexpensive as well as potential electrocatalysts for MOR along with the chance of making catalysts suitable for ORR [8]. Figure 2a, b illustrates the general schematic mechanism of MOR in both acidic and alkaline conditions. In the first step, under acidic conditions, the catalyst adsorbs methanol molecules onto its surface. Moreover, the process of methanol dissociation takes place on the catalyst surface by the cleavage of the C‒H bond. Afterward, the catalyst's surface enables the uptake of H_2_O, leading to the creation of OH_ad_ species. In the fourth step, the C‒H bond undergoes oxidation, leading to the creation of the CO species, which serves as an intermediate. The OH_ads_ radical subsequently undergo additional oxidation of the CO species, resulting in the formation of a CO_2_ molecule. Similiarly in alkaline media, during the initial stage, the catalyst's surface undergoes adsorption of both the methanol molecule and OH^−^ species. After the dissociation of methanol, the catalyst generates several carbonaceous intermediates [9, 10]. After that, OH_ads_ and OH^−^ (which are formed when surface-adsorbed H_2_O is activated), oxidize these intermediates and CO_2_ molecules are formed during the oxidation process. Furthermore, in very acidic environments, the presence of CO intermediates can adversely affect the catalyst's active sites, particularly Pt, [11, 12] resulting in decreased efficiency. Conversely, in a simple setting, intermediate chemicals, like CO, can be easily oxidized due to the heightened reactivity of OH^−^ species. As a result, the catalyst is less susceptible to inefficiency [13–15].

Full methanol oxidation in acidic as well as basic electrolytes is a six-electron process to proceeds as follows (Eqs. 1, 2) [17];

1 $${\text{CH}}_{3} {\text{OH}} + {\text{H}}_{2} {\text{O}} \to {\text{CO}}_{2} {\text{ + 6H}}^{ + } + 6{\text{e}}^{ - } \quad E^{0} = 0.02\;{\text{V;}}\;{\text{pH}} = 0$$

2 $${\text{CH}}_{3} {\text{OH}} + 6{\text{OH}}^{ - } \to {\text{CO}}_{2} {\text{ + 5H}}_{{2}} {\text{O}} + 6{\text{e}}^{ - } \quad E^{0} = - 0.81\;{\text{V;}}\;{\text{pH}} = 14$$

Nevertheless, MOR utilizes both indirect and direct CO routes instead of the 6e^−^ procedure. The initial stage of the MOR process entails the adsorption of methanol onto the catalyst's surface [9, 18]. This is followed by either the breaking of the C‒H bond through dehydrogenation or the formation of adsorbed methoxy (CH_3_O) through dehydrogenation, which occurs by removing acidic hydrogen. Because the C‒H bond breaks, hydroxy-methyl (CH_2_O)_ads_ are made, which in turn causes the production of CO_ads_ through an indirect process [19]. When the O‒H bond breaks down, it can lead to the formation of (CH_3_O)_ads_ and formaldehyde, which either desorb into the solution or undergo further deoxidation. The methanol oxidation reaction (MOR) on platinum (Pt) catalysts considered CO and formate as stable intermediates. These intermediates are considered to influence the efficiency and reaction kinetics of MOR. Regarding MOR, it appears that CO_ads_ remain stable at low potential within the temperature range of 25–75 °C. This stability can obstruct the active sites of Pt, reducing overall reaction efficiency [20, 21]. Researchers discovered that incorporating Ru with Pt significantly enhances MOR activity, thereby preventing this problem. This is because Ru assists in the oxidation of CO intermediates, which reduces the risk of CO poisoning [22–25].

## Pt-group metals-based materials for methanol oxidation reaction

**Fig. 3:**
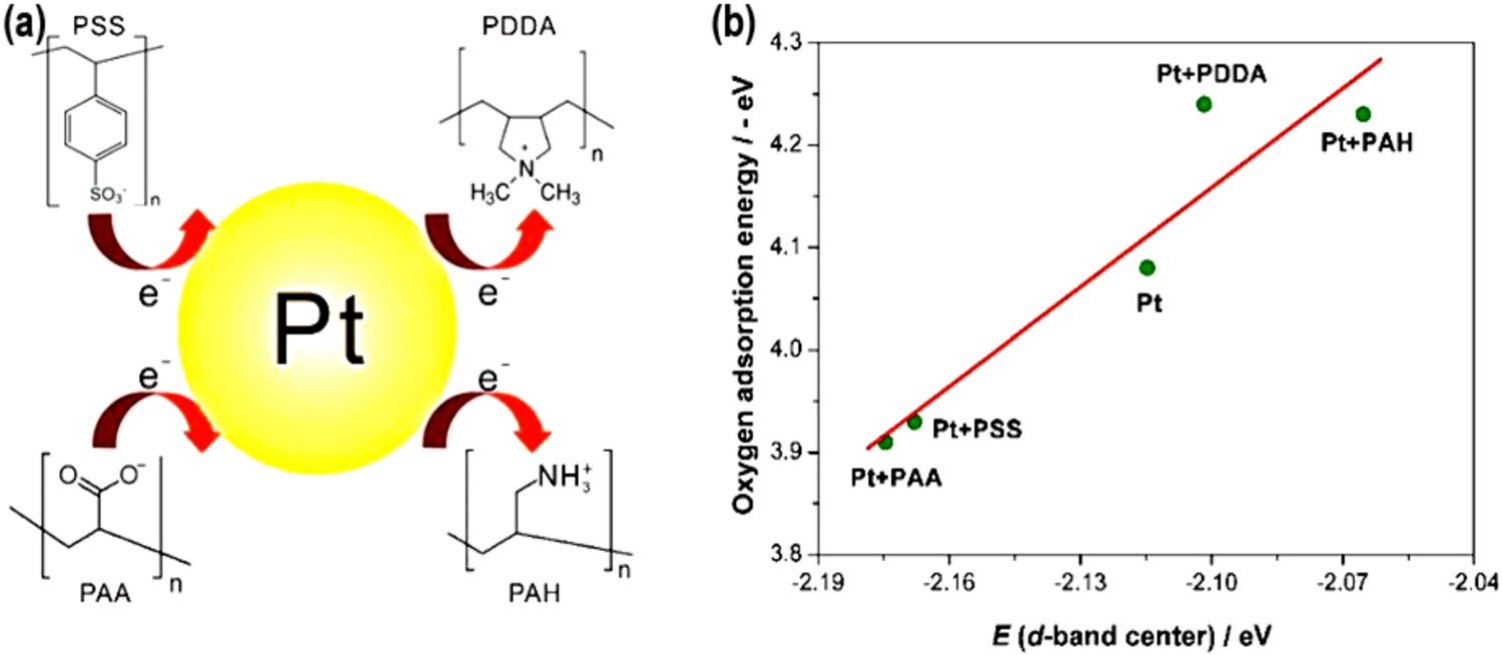
**a** The diagram demonstrates the electrical properties of Pt nanoparticles (Pt NPs) undergo modification by introducing polyanion (PSS) and polycation (PAA, PAH) functional groups, which act as the electron donor–acceptor. **b** This illustration displays the relationship between the d-band center and the energy required for O_2_ adsorption on different Pt slabs. Adapted with permission [39]. Copyright © 2022, Elsevier

Research has shown that the effectiveness of catalysts developed from Pt-group metals (PGM) based materials in alcohol oxidation depends on the particular type of alcohol fuel utilized. When subjected to electrochemical testing on a Pd electrode in basic media, the rate of oxidation for various alcohols increased in the following sequence: methanol (CH_3_OH) > glycol (C_3_H_8_O_3_) > ethylene glycol (C_2_H_6_O_2_) > ethanol (C_2_H_5_OH) > isopropanol (C_3_H_8_O) > n-propanol (C_3_H_8_O). On the Pt electrode, the order of reactivity was as follows: methanol (CH_3_OH) > n-propanol (C_3_H_8_O) > ethylene glycol (C_2_H_6_O_2_) > glycol (C_3_H_8_O_3_) > ethanol (C_2_H_5_OH) > isopropanol (C_3_H_8_O) [26]. The distinct association between adsorption energy and the d-band centers of Pd and Pt metals explains the varying activity trends observed on these metals [27–30]. When it comes to the interaction between adsorbates and the surface, many molecules and atoms exhibit distinct behaviors. For instance, molecules that include robust π-bonds, such as CO or NO, exhibit a unique mode of interaction in contrast to simpler atoms like H or O. This interaction has an independent effect on the adsorption energy, regardless of the d-band center. As the amount of adsorbates on the surface increases, the interactions between them, whether they repel or attract each other, can greatly alter adsorption energy. This change is not directly related to the d-band center [29, 31]. During the adsorption process, it is common for a charge transfer to occur between the adsorbate and the metal surface. This transfer may involve the metal d-states donating charge back to the adsorbate. The d-band center does not exclusively govern the magnitude and orientation of this electron transfer. Hence, the intricate and multifaceted nature of adsorption events constricts the d-band center's ability to make accurate predictions, despite its usefulness as a theoretical tool for understanding patterns in catalytic activity and adsorption features. For accurate predictions, a thorough analysis of detailed computer models (like density functional theory, DFT) is required that consider the entire electrical structure, the shape of the surface, and how each adsorbate interacts with it. Antibonding is observed above the fermi level, while a substantial bonding effect is shown when the d-band center goes higher. The down-shifting of the d-band center [32] creates the perception of a weak connection. Nevertheless, when the electron density fluctuates around the central metal, the d-band center also shifts accordingly [33]. According to this concept, electrocatalysts that have been designed and produced show enhanced electrocatalytic activity [27]. For instance, the Pt–Sn electrocatalyst undergoes a downward shift in the d-band center of Pt due to the chemo-sorption energy with CO-intermediate. This occurs because Sn transfers electrons to Pt, which is a result of a change in electronegativity. Co and Ni are added to Pt–Co and Pt‒Ni electrocatalysts to decrease the displacement of the d-center of Pt. This, in turn, reduces the chemisorption energy with OH species and increases the number of active sites for ORR, MOR, and EOR [34–36]. According to reports, the N-dopant is known to donate electrons to the carbon lattice, increasing the fermi level and charge transfer rate in ORR [37]. The researchers in this study discovered that adding Pt–Rh nanoparticles, tungsten nitride, and carbon nanotubes (CNTs) together improved the catalytic performance and made it less likely that CO would poison the process for EOR. The synergistic effect of tungsten nitride and the nitrogen-doping effect on CNTs are responsible for this improvement [38]. The variation in the loading catalyst step affects the catalytic activity of CNTs, electrocatalysts anchored with charge functional groups. Figure 3a, b shows the impact of constructed polyelectrolytes (PEs) on the surface of CNTs on the charge transfer process and electrocatalytic activity of supported Pt NPs, as reported by Wang et al. [39] Both theoretical analysis and experimental observations indicated that the charged functional group of polymer electrolytes (PEs) had an impact on the d-band center of Pt NPs, which in turn affected their electrocatalytic activity. To enhance the catalytic performance of Pt NPs for MOR, the negatively charged functional groups of polyanions, such as polystyrene sulphonic acid (PSS) and polyacrylic acid (PAA), caused a change in the d-band center of Pt NPs. However, the catalytic activity towards MOR was diminished when Pt NPs blended with the polycations such as polydiallydimethyl-ammonium chloride or polyallylamine hydrochloride, which exhibited an upward shift of the d-band center.

**Fig. 4:**
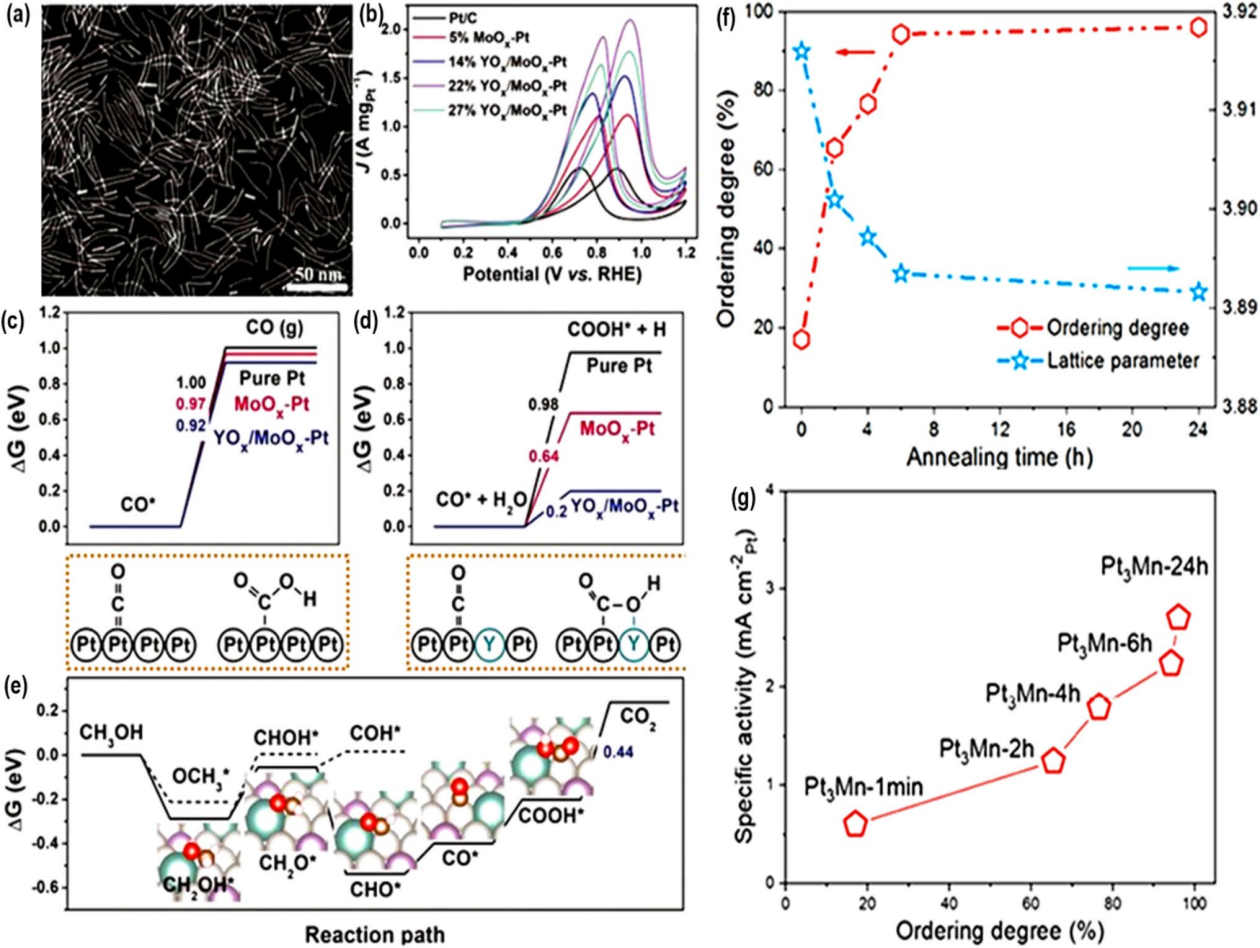
**a** This picture obtained from a high-angle annular dark-field scanning transmission electron microscope (HAADF-STEM) shows very thin nanowires made of 22% YO_x_/MoO_x_-Pt. **b** Illustration of mass-normalized cyclic voltammograms (CVs) of platinum (Pt). **d** The study investigates the process of adsorption of CO* and COOH* on surfaces made of pure Pt and Pt surfaces adorned with YO_x_ (MoO_x_). (**c**–**d**) The theoretical studies investigated the process of adsorption of *CO and COOH* on surfaces made of pure Pt and Pt surfaces adorned with YO_x_(MoO_x_). **e** This diagram illustrates MOR's catalytic process on a YO_x_/MoO_x_-Pt surface, displaying the energy changes involved. Adapted with permission [40]. Copyright © 2021, Wiley–VCH GmbH. The association between the annealing time and the ordering degree/la ice parameter of Pt_3_Mn catalysts is demonstrated in (**f**). **g** Investigating the correlation between the specific activity at 0.8 V and the degree of ordering in Pt_3_Mn. Adapted with permission [41]. Copyright © 2022, American Chemical Society

Furthermore, ultrathin nanowires, with a diameter of less than 2 nm, are well recognized as highly intriguing catalysts for MOR. This is because they possess a greater number of exposed surface-active areas. For instance, Li et al. synthesized ultrathin nanowires made up of 22% YO_x_/MoO_x_-Pt. As depicted in Fig. 4a–e [40], the nanowires exhibited a remarkable mass activity for MOR of 2.10 A mgPt^−1^ and a specific activity of 3.25 mA cm^−2^. Researchers have proposed the presence of a decoupling mechanism between adsorbed CO_ads_ and COOH_ads_. In this process, the catalyst has adsorbed an intermediate species called COOH_ads_, in which the carbon atom is bonded to Pt and the oxygen atom is bonded to an oxophilic element Y. The energy change for the oxidation of CO_ads_ to COOH_ads_ reduces considerably when compared to pure Pt.

In their research, Zhang et al. found that the kinetics of MOR reaction can be fasten by adding void structures to the grain boundaries of Pd_4_Sn wavy nanowires [29]. The numerous flaws present on the surface of Pd_4_Sn-wavy nanowires create additional possibilities for the adsorption of small molecules. Thermodynamically, the presence of a surface void structure enhances MOR's catalytic activity in the HCOOH (HCOO_ads_) pathway, as opposed to the pathway that leads to CO production. Moreover, the relaxing of the local environment at Pd sites offsets the synergistic effect of significant structural deformation around Sn sites. Chen et al. [41] conducted another study to examine the impact of the annealing period on the MOR (methanol oxidation reaction) activity of L_12_-phased Pt_3_Mn@Pt skin. During the annealing process, the degree of order in the Pt_3_Mn intermetallic core increases, and the lattice parameter decreases as the duration of annealing goes from 1 min to 24 h, as shown in Fig. 4f, g [41]. The presence of a Pt_3_Mn core induces an increasing compressive strain in the Pt outer layer, resulting in a constant reduction in the distance between adjacent Pt atoms. Following a 24-h annealing process, they successfully produce the most efficient catalyst, exhibiting a specific MOR of 2.71 mA cm^−2^ and a mass activity of 1.98 AmgPt^−1^. Further mathematical studies demonstrate that the augmentation in the MOR activity of Pt_3_Mn@Pt catalysts is mostly attributed to compressive strain rather than the influence of the ligands [42].

## Non-Pt-group metals based electrocatalysts for methanol oxidation reaction

It would be ideal to create new catalysts that are long-lasting, have high catalytic activity, anti-CO capabilities, and are easy to make if we want to catalyze MOR at a practical level. Consequently, efforts are being made to create catalysts for direct methanol fuel cells (DMFCs) that need a reduced number of noble metals. Within this framework, co-oxides are gaining popularity as HOR catalysts due to their exceptional inherent properties, including electrical and structural flexibility, tunable chemical properties, and redox rich chemistry. Anodic deposition was used by Jafarian et al. [43] to construct a glassy carbon (GC) electrode modified with Co(OH)_2_. Then, the electrode was examined for activity in the methane oxidation process (MOR). The catalytic reaction demonstrated that cobalt exists in several valence states, with the Co(IV) state serving as the MOR active site. Zafeiratos [44] went a step further by studying how spinel Co_3_O_4_ and rock salt CoO's structures affected their HOR activity. Based on the data, spinel Co_3_O_4_ could start partial MOR by converting CH_3_OH into HCHO because it has a more mobile O-lattice. Another study found that Xia et al. [45], created a 3D mesoporous Co_3_O_4_ with improved catalytic activity. The enormous surface area of this catalyst (118–121 m^2^/g) allowed it to exhibit significantly higher MOR activity in comparison to non-porous Co_3_O_4_.

**Fig. 5:**
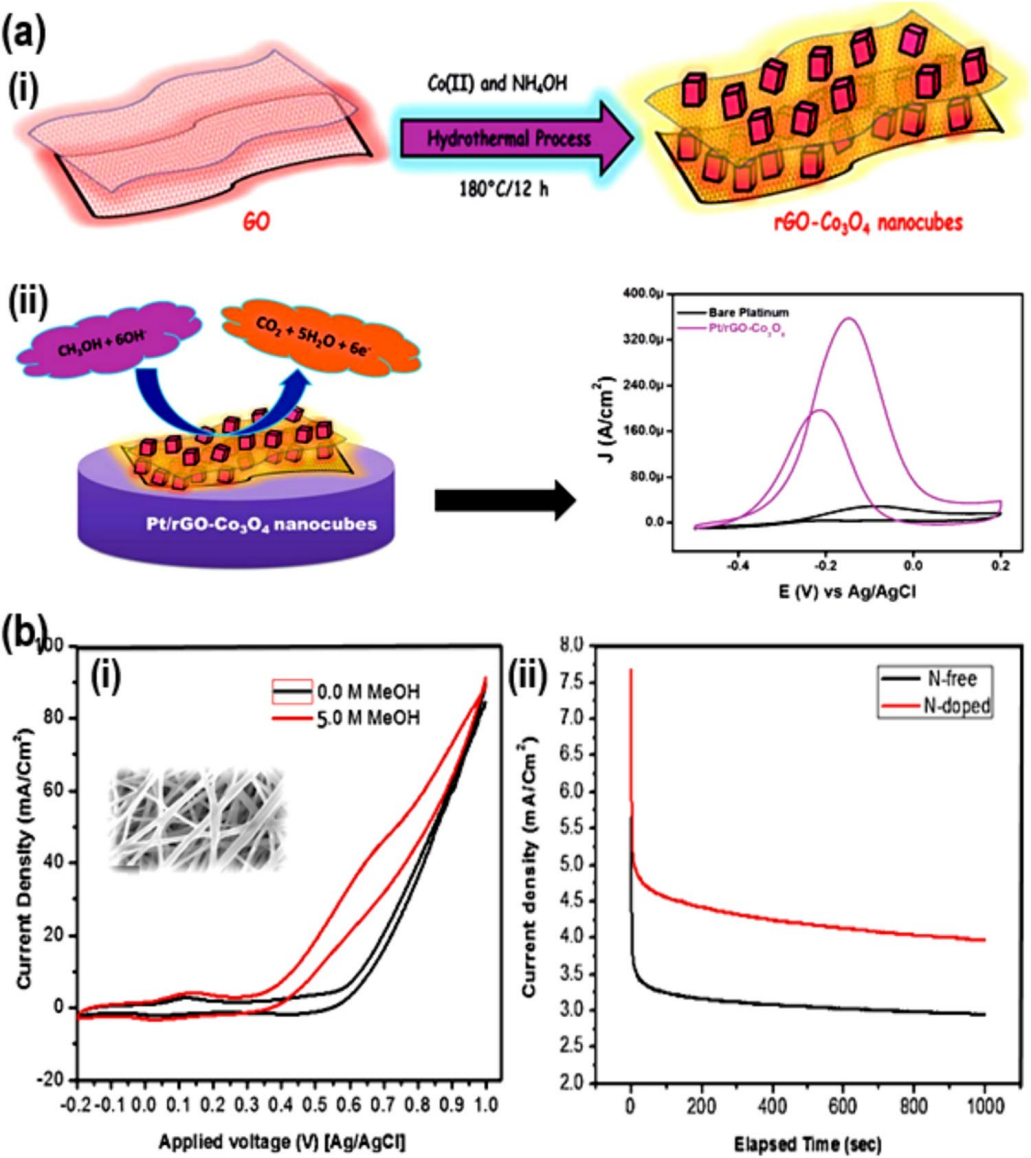
**a** (i) Preparation protocols, and (ii) MOR pathway for the Pt/rGO–Co_3_O_4_. Adapted with permission [46]. Copyright @2014, The Royal Society of Chemistry. **b** (i) CVs, and (ii) Chronoamperometric curves, recorded in 1.0 M KOH + 3.0 M CH_3_OH solution, for Co/N-CNFs. Adapted with permission [47]. Copyright © 2015, Elsevier

**Fig. 6:**
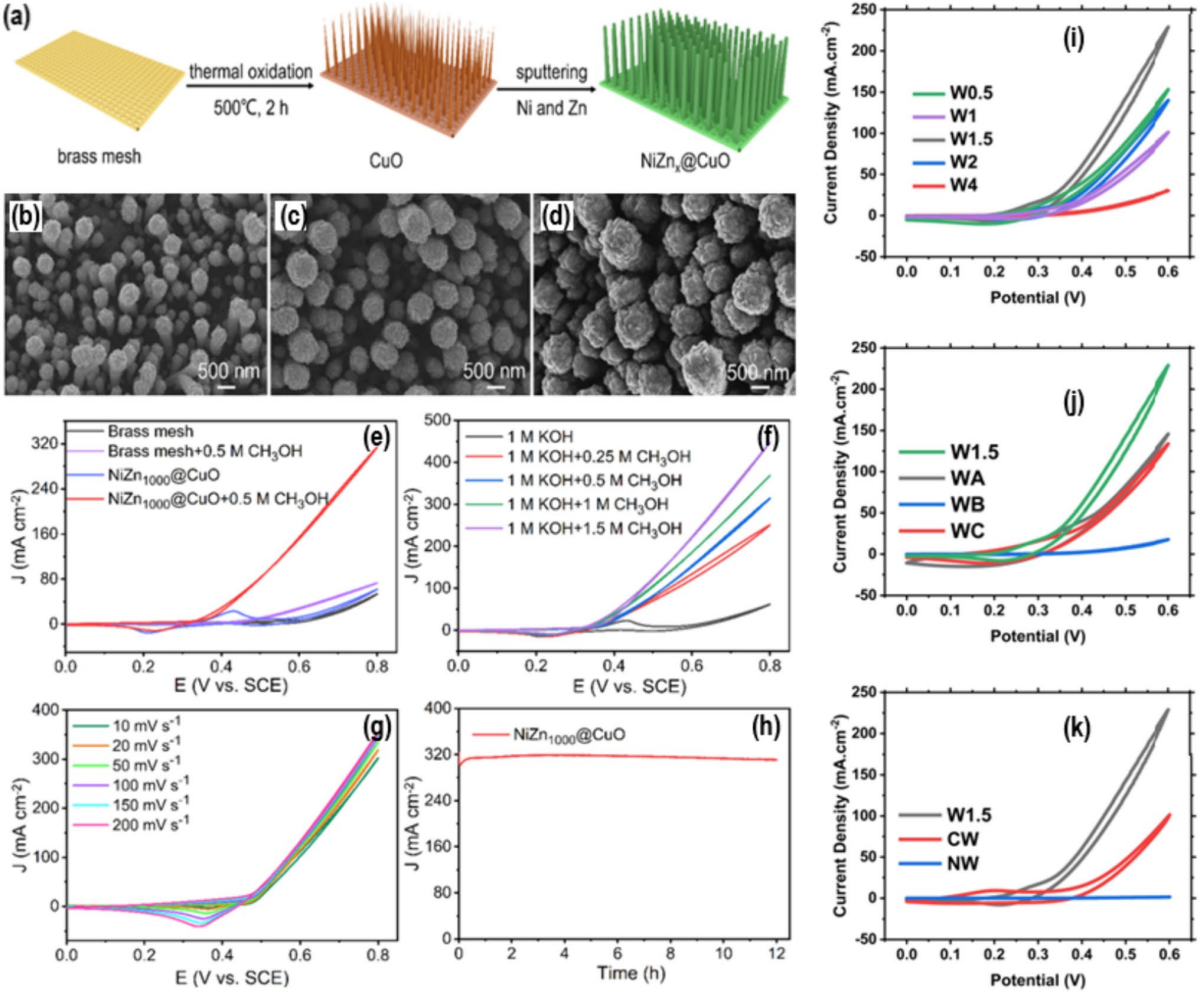
**a** The diagram illustrates the process of creating the NiZn_x_@CuO nanoarray structures. The obtained pictures are SEM views of NiZn_x_@CuO nanoarray designs. These architectures consist of NiZn alloy sheets with varying thicknesses: **b** 1000 nm, **c** 1500 nm, and **d** 2000 nm. The CVs of the NiZn1000@CuO and brass mesh catalysts in a KOH solution are shown, with and without 0.5 M methanol. The scan rate used was 50 mV s^−1^. The CVs of the NiZn1000@CuO catalyst in a KOH solution containing methanol concentrations of 0.25 M, 0.50 M, 1.00 M, and 1.50 M. The scan rate used was 50 mV s^−1^. The CVs of NiZn1000CuO were measured in a 1 M KOH solution containing 0.5 M methanol at different scan rates. **h** A chronoamperometry experiment was conducted on the NiZn1000@CuO catalyst at a potential of 0.80 V for a duration of 12 h. Adapted with permission [48]. **e** The CVs of the NiZn1000@CuO and brass mesh catalysts in a KOH solution are shown, with and without 0.5 M methanol. The scan rate used was 50 mV s^−1^. **f** The CVs of the NiZn1000@CuO catalyst in a KOH solution containing methanol concentrations of 0.25 M, 0.50 M, 1.00 M, and 1.50 M. The scan rate used was 50 mV s^−1^. **g** The CVs of NiZn1000CuO were measured in a 1 M KOH solution containing 0.5 M methanol at different scan rates. Copyrights © 2023, ACS. **i**–**k** The graph depicts the comparison between different sample performances in 1 M KOH + 1 M methanol. Adapted with permission [25]. Copyrights© 2024, RSC

A different picture, Fig. 5ai, shows how Shahid et al. [46] made a mix for metal–organic frameworks (MOR) and used nanocarbons like rGO to make Co_3_O_4_ more conductor. This catalyst greatly increased the MOR activity compared to bare Co_3_O_4_, rGO, and even the typical Pt catalyst. As shown in Fig. 5aii, the catalyst variant with 2% rGO was the best, showing a peak current density of 362 mA/cm^2^ during MOR. In order to make a catalyst that conducts electricity better, Thamer et al. [47] used an electrospinning method to make N-doped carbon nanofibers (Co/N-CNFs). These nanofibers were very good at absorbing things and didn't block electrons from moving through them. The sample that hasn't been doped (63.56 mA/cm^2^) in Fig. 5bi–ii has the most activity for Co/N-CNFs at 4% N-content. This is because urea was used as an N-source from 0 to 5% to study how N-content affects the material. In a different study, Han et al. [48] made highly ordered NiZnx@CuO nanoarray structures by heating them up and then using magnetrons to deposit them on a brass mesh base Fig. 6a–h. The researchers discovered that these nanoarrays were highly efficient electrocatalysts for the MOR process. By utilizing different preparation methods for NiZn@CuO nanoarray catalysts, the researchers also discussed about their catalytic activity as electrocatalysts. They discovered that the NiZn1000@CuO electrode, which had been heated to 500 °C for two hours and NiZn alloy film showed a thickness of 1000 nm with a high current density of 449.3 mA/cm^2^ at 0.8 V for MOR in alkaline media. Furthermore, this electrode exhibited exceptional operational stability, maintaining a retention rate of 92% even after 12 h. The catalysts well-organized hierarchical structure enhances electrocatalytic activity through providing numerous active sites and a synergistic effect, which contributes to its outstanding MOR performance. Additionally, this catalytic structure also promotes efficient mass and electron transfer. It was impressive to show how fractional Zn atoms could be dissolve from the NiZn alloy. This caused the formation of uneven surface nanorods and also increased their specific surface area. The results indicate that the NiZn1000@CuO nanoarray structure has the potential to serve as a viable substitute for Pt-group metals. It has the potential to effectively function as an anode catalyst for DMFCs while also being cost-effective and durable. Mahmoud et al. [25] in another study, use transition metals as the basis for their investigation and present a non-precious group metal (non-PGM) electrocatalyst. (Fig. 6i–k). This electrocatalyst exhibits significant promise in enhancing the efficiency of direct methanol fuel cells (DMFCs). They used a co-precipitation method with different amounts of nickel, cobalt, and tungsten to make a nickel–cobalt mixed tungstate compound. They also tested and validated the materials using a range of characterization techniques. The SEM investigation revealed that the materials contain agglomerated amorphous random circular nanocomposite structures. The electrocatalysts exhibited superior electrochemical performance, with the nanocomposite with a Ni:Co:W ratio of 1:1:1.5 (W1.5) demonstrating the highest performance. When methanol was oxidized at a scan rate of 50 mV s^−1^ in a 1 molar methanol solution at a potential of 0.6 V, the composite material had a higher current density of 229 mA cm^−2^. In addition, it exhibited the lowest onset potential of 0.33 V. The results demonstrate the identification of a durable, inexpensive metal material that is suited for direct methanol electro-oxidation procedures. The finding indicates the possibility of creating affordable and eco-friendly electrocatalysts as a replacement for expensive catalysts that are commercially available in the field of catalysts [49, 50].

**Fig. 7:**
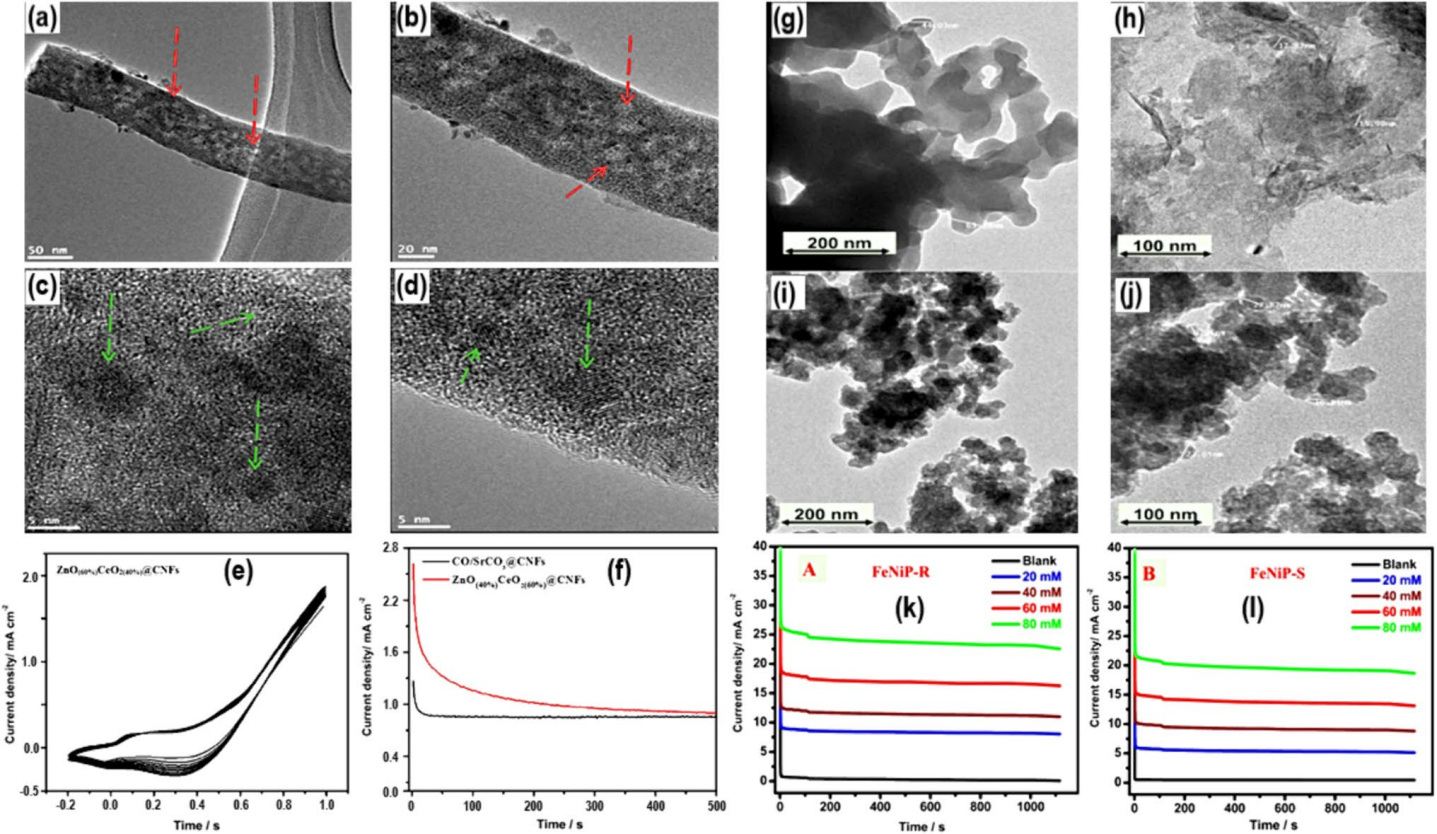
**a**–**d** The diagram depicting the TEM images that are recorded at different magnifications for ZnO_(x)_CeO_2(1-x)_ catalyst, **e** CV, and **f** Illustration of chronometric curves that are recorded in methanol containing electrolyte, for the prepared catalysts. Adapted with permission [51]. Copyright © 2015, Elsevier. **g**–**j** The diagram depicting the TEM images that are recorded at different magnifications for FeNi-based material, **g–l** Illustration of chronometric curves for the prepared FeNiP-R, and FeNiP-S catalysts. Adapted with permission [52]. Copyrights © 2022, MDPI

Ghouri et al. [51] evenly spread ZnO_(x)_CeO_2(1−x)_ nanodots on carbon nanofibers using a template-free method and used it as anode catalysts for the electrooxidation of methanol. The characterization methods revealed that the material consists of carbon nanofibers decorated with ZnO and CeO_2_ as depicted in Fig. 7a–f. The researchers employed cyclic voltammetry (CV) on a glassy carbon electrode modified with ZnO_(x)_CeO_2(1-x)_ nanodots on carbon nanofibers to comprehensively investigate the electrochemical oxidation of methanol in alkaline solutions. They conducted a comprehensive analysis to investigate the electrocatalytic oxidation of methanol by altering its concentration. The ZnO (40%) and CeO_2_ (60%) nanodots exhibited current densities of 5.3 and 16.3 mA/cm^2^ at CNFs. When ZnO (40%) and CeO_2_ (60%) nanodots were used on CNFs, the onset potential was − 50 mV lower than when Ag/Ag was used. They consider this value to be higher than that of other non-precious electrocatalysts that have been characterized. In another study, Khalaf et al. [52] conducted another investigation where they created a novel phosphate substance consisting of two metals (Fe and Ni) (Fig. 7g–j). The developed material has shown effectiveness in MOR activity, as illustrated in Fig. 7k–l. The characterization techniques validate that the sol–gel process has shown greater ability to generate nanoscale particles with increased agglomeration, in contrast to the reflux procedure. The electrochemical measurements showed a substantial increase in the current values obtained from both electrodes, FeNiP-R and FeNiP-S. During the electrooxidation of MeOH using FeNiP-S, the current density increased from 0.14 mA/cm^2^ at 0.402 V to 2.67 mA/cm^2^ at 0.619 V. This corresponds to an almost 109-fold rise in comparison to the present density measurement of 0.0243 mA/cm.^2^ at 0.62 V in the absence of MeOH [53]. It was found that the FeNiP-R electrode was better at electrocatalysis than the FeNiP-S electrode at all methanol concentrations up to 80 mmol/L. The increase in anodic current density and charge transfer resistance suggests that the methanol electrooxidation process is occurring on the specially designed Fe/Ni-phosphate bimetallic catalyst [54–56]

## Atomically precise single-atom catalysts for methanol oxidation reaction

**Fig. 8:**
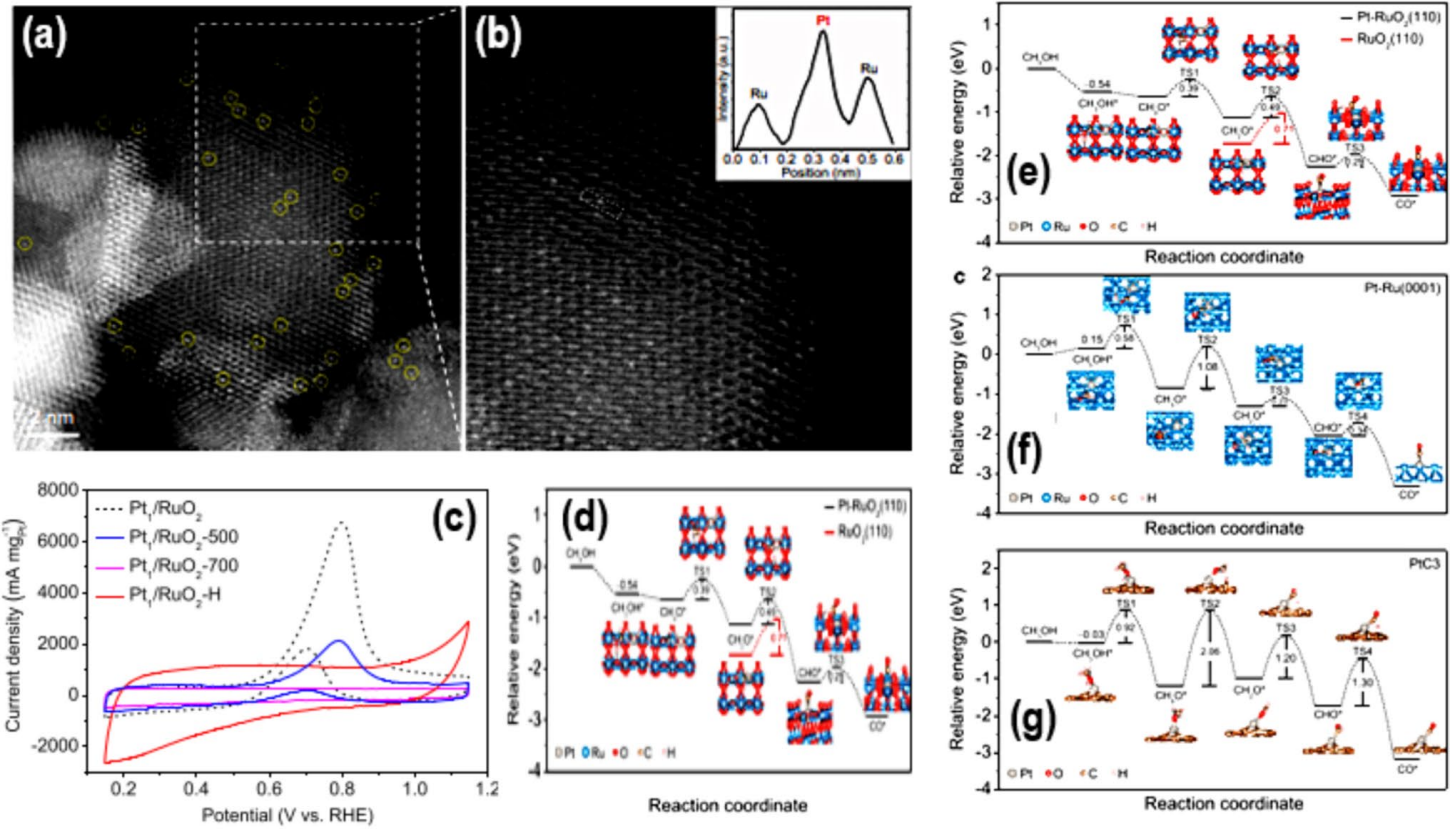
**a**, **b** HAADF-STEM images, **c** CV curves, and **d**–**f** Diagram illustrating calculated reaction free energy curves of samples. **g** CO energy barriers, for the prepared Pt-RuO_2_ samples. Adapted with permission [69]. Copyrights © 2021, Springer Nature

Environmental conditions and the geometric arrangement of Pt greatly influence its electrocatalytic performance [57]. Researchers have thoroughly investigated two methods to boost MOR activity and reduce Pt concentration. Changing Pt's structure and/or shape is the main idea behind it. Examples of this include core–shell Pt combinations [31] or hollow or framed Pt structures [58, 59]. Furthermore, combining Pt with Co, Ni, and Sn yields an easily exploitable metal. Unfortunately, the catalysts have poor mass activity because they contain Pt in the form of NPs larger than 1 nm. Furthermore, it is well established that CO_ads_ in MOR predominantly act as a toxin for Pt NPs, leading to a decrease in their activity [60]. It is therefore very important, both theoretically and practically, to make new electrocatalysts for MOR that are based on Pt, work better, and are less likely to poison. Researchers have discovered a new group of single-atom catalysts (SACs) [61–63]. These can help with many electrocatalytic tasks, including alcohol oxidation reactions and reduction reactions for oxygen, carbon dioxide, and nitrogen. The use of Pt is very efficient in Pt SACs, which can greatly speed up the oxidation of carbon monoxide. In MOR, the electrochemical dehydrogenation of methanol to CO necessitates the presence of three adjacent Pt atoms. A single Pt-atom on a CNTs forms SACs [64, 65]. The atomic arrangement significantly influences the catalytic efficiency of each individual atom [66]. Previous studies have shown that changing the connection between the individual atom and substrate can alter the electrical configuration and arrangement of the central individual atom [67, 68]. For instance, Zhiqi Zhang et al. [69], used an easy adsorption-impregnation method to successfully attach single Pt atoms to the surfaces of RuO_2_ and carbon black (VXC-72) in their study (Fig. 8a–c). They measured the mass activity of the Pt_1_/RuO_2_ single-atom catalyst. Figure 8c demonstrates that the stability of these SACs against MOR was significantly higher than that of most previously studied Pt-based catalysts. Density-functional theory (DFT) calculations have greatly contributed to our understanding of the MOR's operation. It involves the electrooxidation of CO and the dehydrogenation of methanol. The results shown in Fig. 8(d–g) back up the experimental findings that the prepared SACs can effectively oxidize alcohol. The findings suggest a potential method for utilizing SACs in direct alcohol fuel cell applications [70–72].

**Fig. 9:**
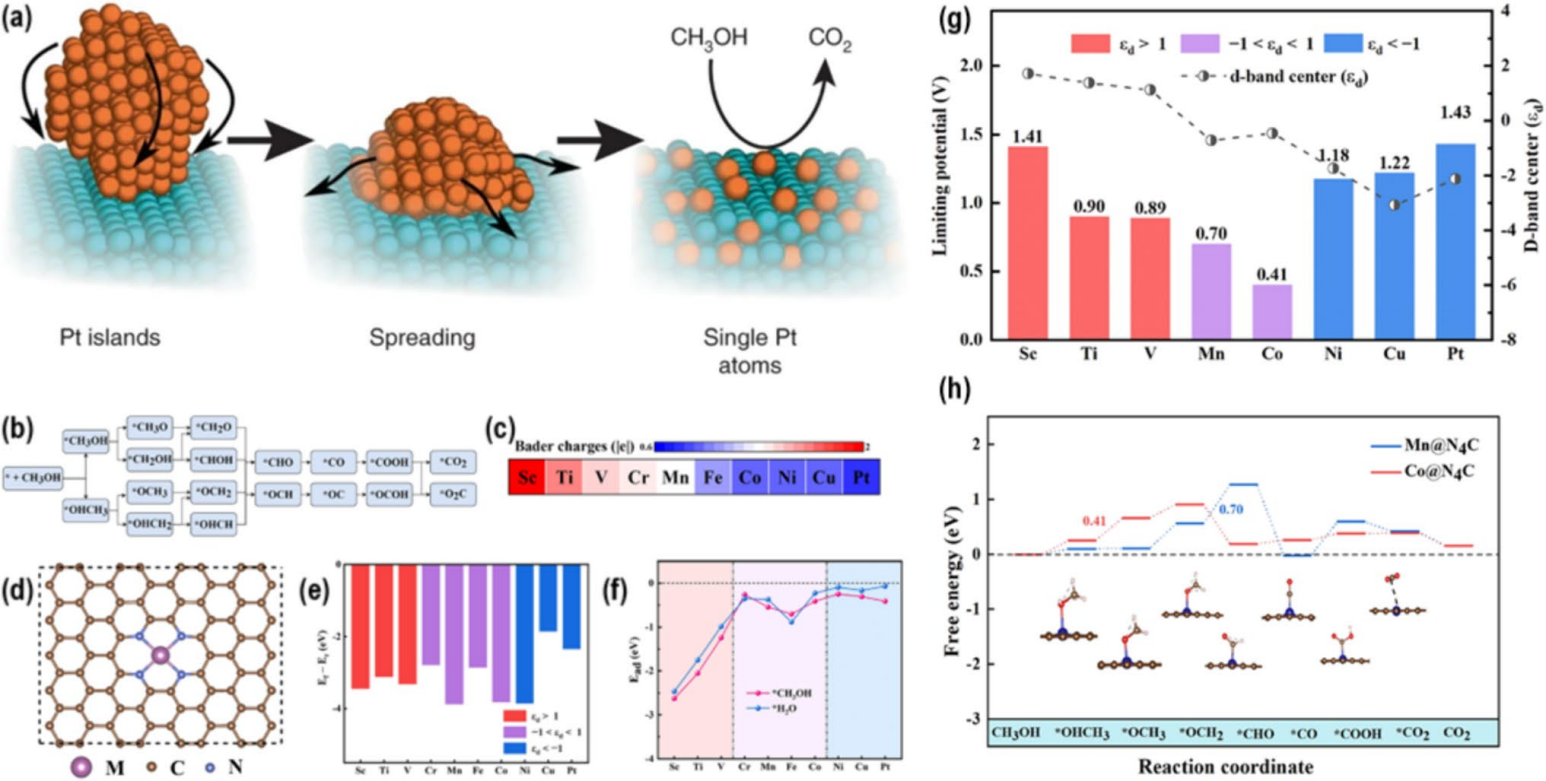
**a** Graphical illustration showing conversion of Pt islands into single Pt atoms and their promising candidature for MOR. Adapted with permission [73] Copyrights © 2022, Nature. **b** Diagram depicting reaction pathways for MOR. **c** Illustration of atomic structures of M@N_4_C. **d** The number of transferred charges from TM atoms to substrate. **e** Depiction of energy difference between E_f_ and E_c_ of M@N_4_C. ε_d_ means the d-band center of transition metal. **f** Adsorption energies of CH_3_OH and H_2_O on M@N_4_C. **g** Limiting potentials and corresponding d-band centers for M@N_4_C. **h** Representation of free energy diagrams of Mn@N_4_C and Co@N_4_C. Adapted with permission [77]. Copyrights © 2023, ACS

Single-atom catalysts are highly efficient due to their high Pt content, which significantly enhances electrocatalytic activity. In addition, PtRu alloy nanoparticles demonstrate superior catalytic activity in the methanol oxidation reaction. To leverage the remarkable reactivity of single Pt atom catalysts supported by highly efficient Ru, it is imperative to address the synthetic challenge associated with achieving single Pt atoms on ultrafine noble metal particles. Regarding this discussion, Poerwoprajitno et al. [73] describe a way to create and spread Pt islands on Ru branching nanoparticles that created single Pt-atoms-on-Ru (Fig. 9a). Using in situ TEM, researchers observed that thermodynamics, particularly the formation of strong Pt-Ru bonds and the decrease in surface energy within the Pt islands, drive the production of a stable single-atom structure. The single-atom platinum atom adsorbed on ruthenium has long-term stability and does not exhibit adverse reactivity with carbon monoxide. This suggests that it has the ability to maintain a high level of current density and mass activity while undergoing the methanol oxidation reaction [74–76].

In another study, Zhang et al. [77] performed a thorough investigation of the activity patterns of electrochemical MOR using DFT calculations. The study focused on a single transition-metal atom embedded in N-coordinated graphene (M@N_4_C), as shown in Fig. 9(b–h). After examining the free energy diagrams of MOR on M@N_4_C, they determined that Co@N_4_C is the most effective catalyst for MOR. This is due to its unique charge transfer and electronic structure result in a low limiting potential of 0.41 V. They conducted an important investigation to figure out how the volcanoes in MOR related to the M@N_4_C catalysts by analyzing the d-band center and Gibbs free energy of G_CH3OH_^*^ and G_CO_^*^ in one and two dimensions, respectively. This study provides theoretical guidelines for improving MOR activity on M@N_4_C and offers insights for developing MOR electrocatalysts that are both effective and efficient.

**Fig. 10:**
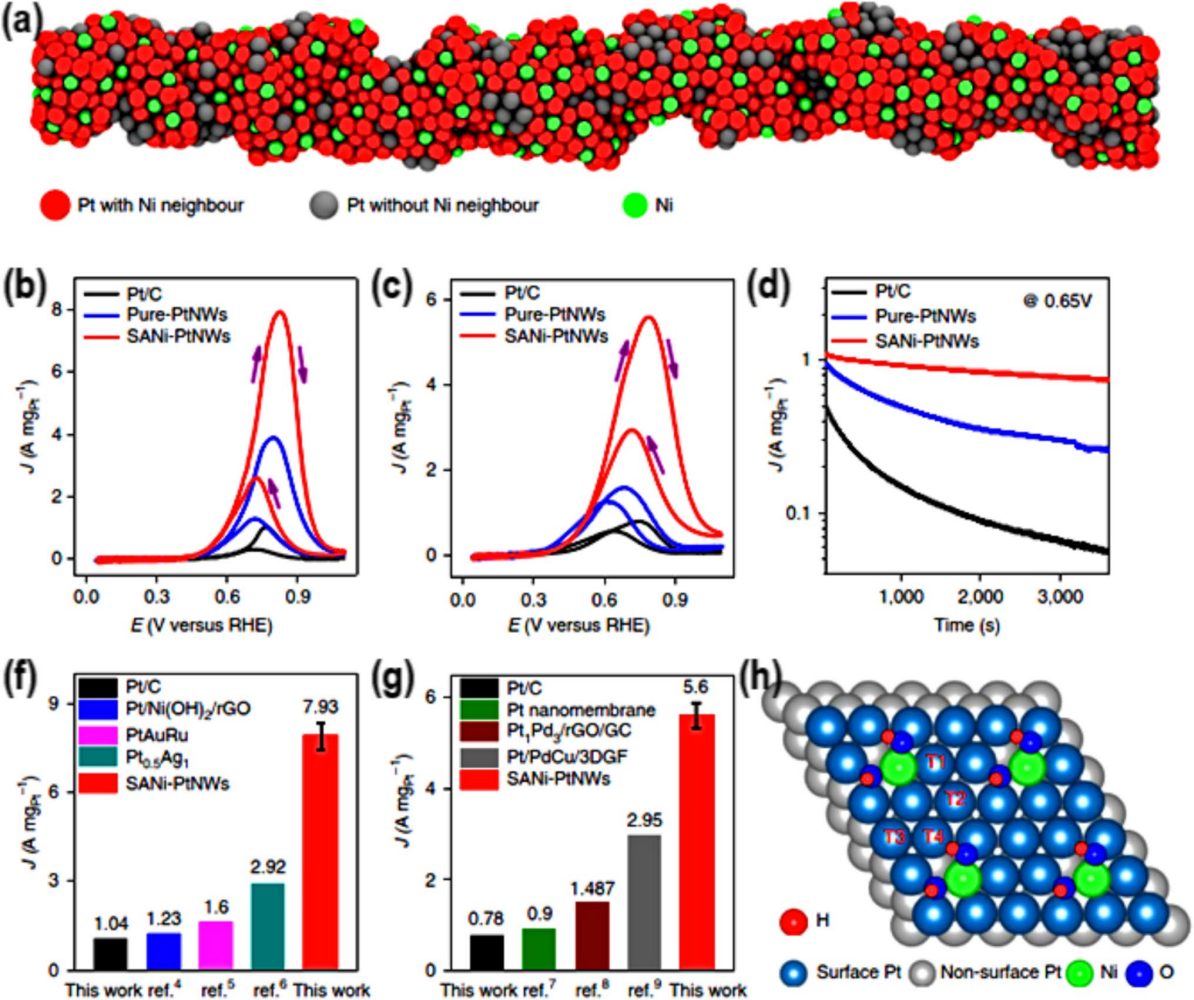
**a** 3D illustration, **b**, **c** CVs for MOR and EOR, **d** MOR Chronoamperometric curves, and **f**, **g** Comparison for the MOR and EOR current density, for the SANi-PtNWs material. **h** Crystal model for the SANi-decorated Pt (111) surface, displaying various active sites for CO adsorption. Adapted with permission [78]. Copyrights © 2019, Nature Publishing Group

In another work, Li et al. [78], also sought to better understand the single atom Ni-Pt nanowire’s (SANi-PtNW) potential as multifunctional electrocatalysts by studying them for MOR and EOR (Fig. 10a). When contrasted with the commercial Pt/C electrocatalyst, the synthetic SANi-PtNW showed higher performance at 3.87 A/mg Pt. The fact that SANi-PtNWs have a lower initial overpotential than Pt/C further supports their lower activation barrier for methanol oxidation on their surface (144 mV). At 1 M ethanol and 1 M KOH, SANi-PtNW outperforms Pt/C for EOR by a factor of 7 and 3, respectively. As shown in Fig. 10(b-g), SANi-PtNW outperforms pure Pt-NWs and Pt/C in terms of long-term dependability in MOR. DFT research revealed all reaction pathways to be exothermic at the experimental potential. SANi-modified Pt top-sites are not as good at absorbing CO_2_ (by 0.06–0.28 eV) as Pt (111) top-sites in the last step, CO_2_ production (Fig. 10f). To alter MOR or EOR activity, further new knowledge may be helpful for single-atom tailoring.

**Fig. 11:**
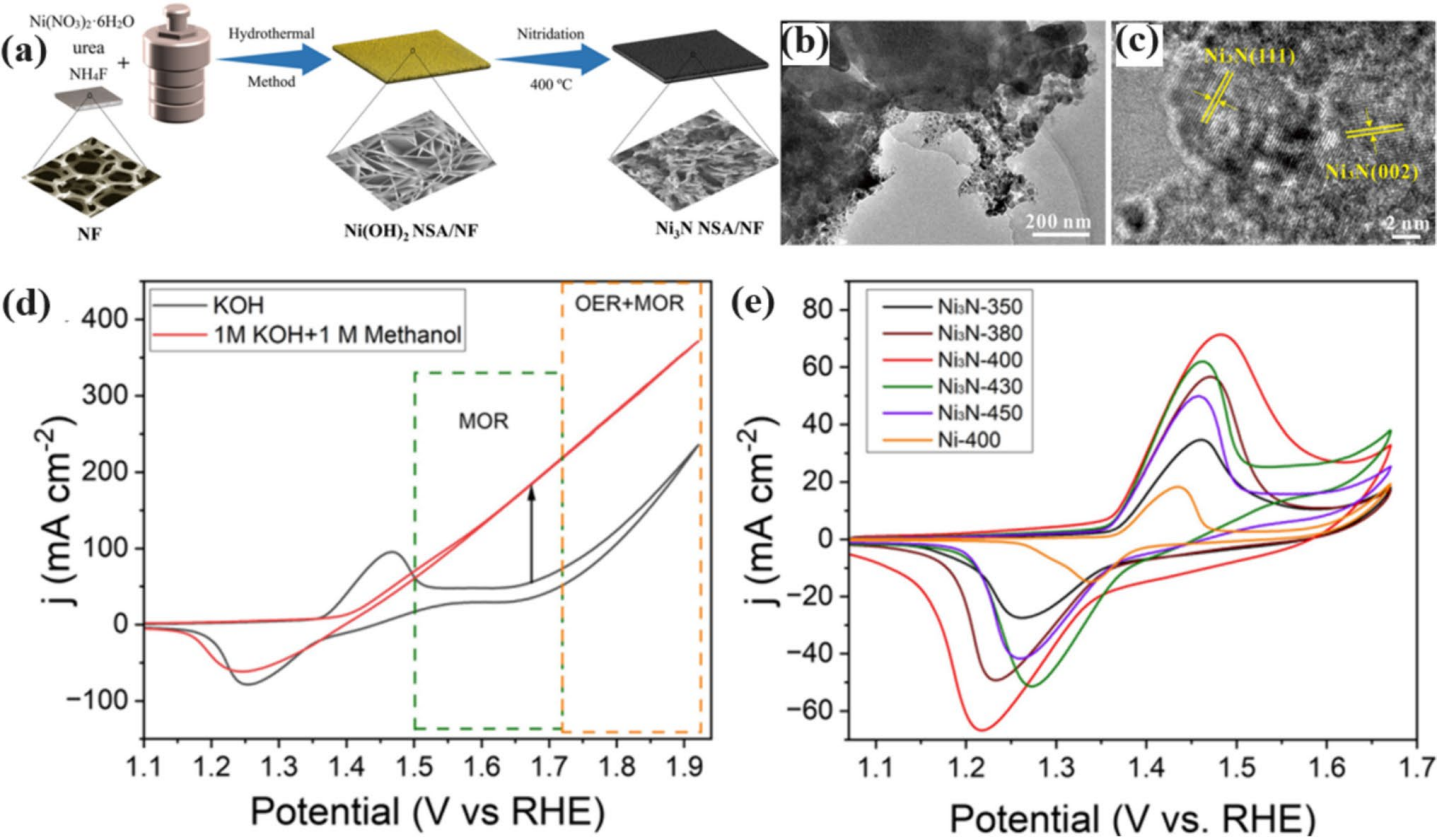
**a** Schematic illustration of the process for developing the Ni_3_N nanosheet arrays on a nickel foam substrate (Ni_3_N NSAs/NF). **b** Transmission Electron Microscopy (TEM) and **c** High-Resolution TEM (HR-TEM) pictures of Ni_3_N-400 **d** CV curves of Ni_3_N-400 in a 1.0 M KOH solution with and without a 1.0 M methanol solution were obtained using a scanning rate of 50 mV s^−1^. **e** The CV curves of Ni_3_N at various temperatures and Ni-400 in 1.0 M KOH were obtained using a scanning rate of 50 mV s^−1^. Adapted with permission [79]. Copyrights © 2023, AIP Publishing

Zhang et al. [79] recently used a hydrothermal method along with nitridation to designed an electrocatalyst for the MOR that works highly efficiently. The process consisted of constructing porous Ni_3_N nanosheet arrays on a nickel foam (NF) substrate. A straightforward two-step technique can create the highly porous Ni_3_N nanosheet arrays, as shown in Fig. 11a. The first step is to employed a hydrothermal method to grow the Ni(OH)_2_ NSAs on a very clean and permeable NF surface, which acts as a conductor. Next, they annealed the Ni(OH)_2_ NSAs/NF nitride at 400 °C, passing NH_3_ gas over it to promote the formation of the Ni_3_N NSAs/NF composite. They further evaluated the Ni_3_N non-stoichiometric nitride semiconductor/nanofiber electrode for revealing its MOR activity. The TEM image (Fig. 11b) clearly demonstrates the abundance of tiny nanoparticles in the Ni_3_N-400 nanosheet. The HR-TEM image of Ni_3_N-400 in Fig. 11c provides a highly detailed view of the lattice fringes. The distance between these fringes, referred to as the interplanar d-spacing, ranges from 0.203 to 0.215 nm. The values are consistent with the hexagonal Ni_3_N crystal planes (111) and (002). They use CV to analyze the electrochemical properties of the newly created catalyst, Ni_3_N-400. Prior to that, they perform a comparative test to assess the characteristics of the MOR. In order to determine the catalyst's range, researchers use CV in a 1.0 M KOH solution, with or without methanol. The potential range observed during this process varies from 1.1 to 1.8 V. Figure 11d displays the MOR characteristics of the Ni_3_N-400 catalyst in a potential range of 1.5–1.7 V. They observe these characteristics following the appearance of the Ni^2+^ oxidation peak, which happens in the absence of the methanol solution. However, OER occurs on the catalyst at around 1.75 V compared to the RHE, requiring a substantial excess voltage. This indicates the catalyst exhibits superior performance at a lower MOR potential in comparison to OER. As a result, they define a voltage range of 1.1–1.7 V as appropriate for evaluating the MOR activity of Ni_3_N-400. Despite the significant formation of NiOOH in methanol oxidation, they subject all samples to CV testing. The tests involve 50 cycles performed at a scan rate of 50 mV s^−1^ in a 1.0 M KOH solution. Initially, they treated the samples to increase the thickness of the electrocatalytic layers and promote the growth of Ni(OH)_2_/NiOOH on the surface [44, 45]. As depicted in Fig. 11e, all of the catalysts exhibit two redox peaks that correspond to the Ni^2+/^Ni.^3+^ redox reaction. Ni_3_N-400 is the catalyst that stands out due to its superior current density for both oxidation and reduction peaks. This implies that there are a greater number of active sites available to interact with a 1.0 M KOH solution [46].

## Molecular catalysts for MOR

**Fig. 12:**
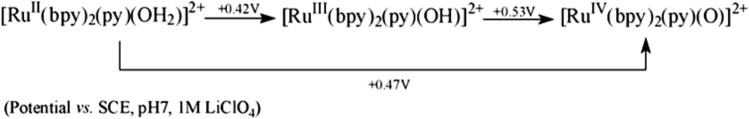
Mechanism of metal oxo-complexes towards alcohol oxidation. Adapted with permission [82]. Copyright @1989, Elsevier

Methanol oxidation electrochemically is very important due to its close relation to fuel cell and energy research. The extensively utilized molecular catalysts for alcohol oxidation are complexes based on Ruthenium (Ru) and Nickel (Ni) [71, 80]. Besides, Ru and other metal oxo-complexes are also used as suitable oxidizing agent for alcohol [81]. Figure 12 illustrates the proton couple electron transfer by the high valent Ru (IV)-oxo species being generated from corresponding Ru (II)-aquo-complex.

**Fig. 13:**
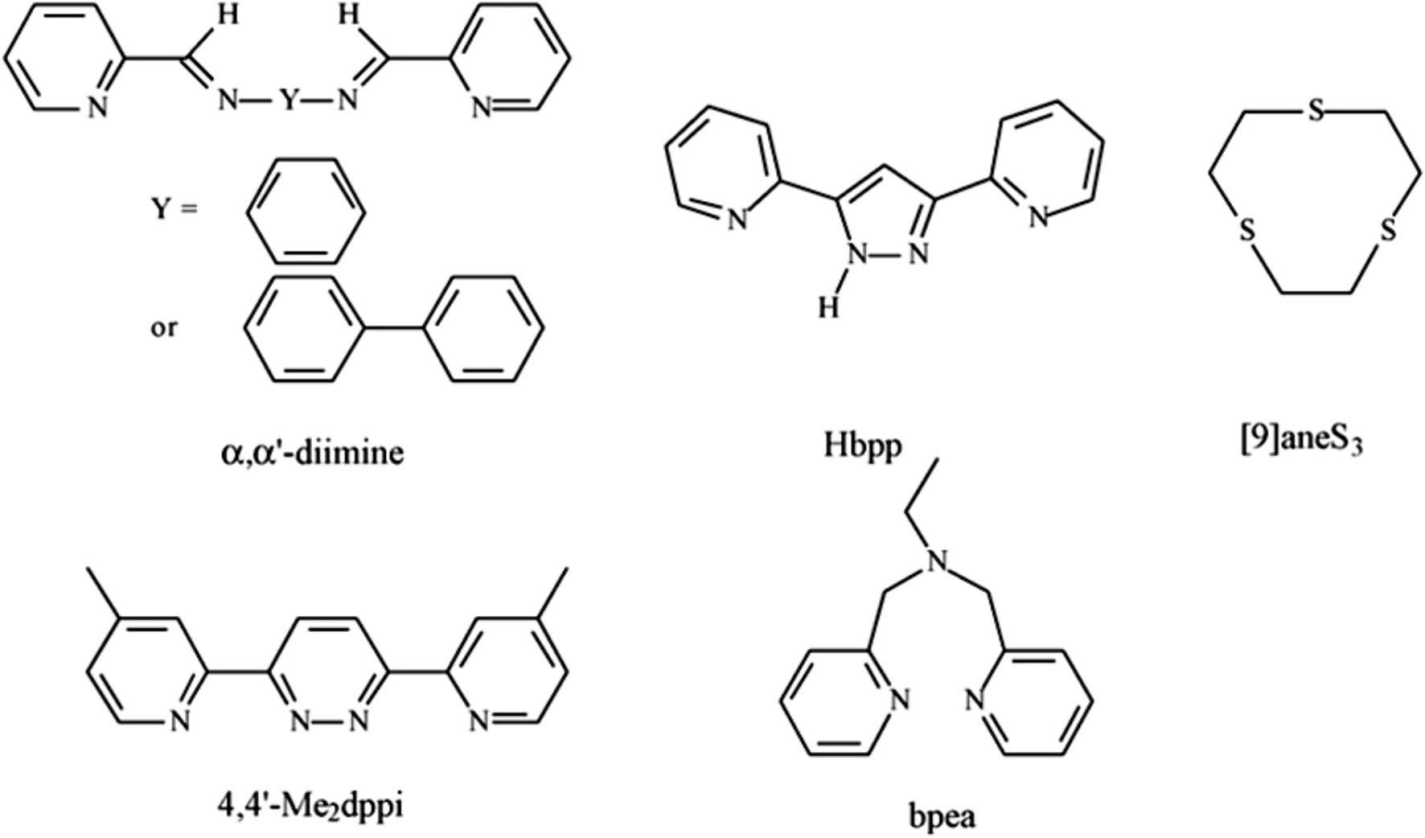
Structure of various ligands [84]

There have been numerous recent reports on novel Ru-oxo compounds that incorporate diamines and modified polypyridyl ligands (Fig. 13). In this study, Lahiri et al. [83] presented evidence of the oxidation process of Ru-aquo species [Ru(tpy)(2,3′-di-imine)(H_2_O)]^2+^ (α,α′- di-imine = NC_5_H_4_C(H) N(C_6_H_4_)nNH_2_, n = 1 and 2). Ru-oxo acts as a catalyst for the oxidation of benzyl alcohol, resulting in the formation of benzaldehyde [83]. Ru-oxo catalyzes the oxidation of benzyl alcohol to benzaldehyde. Hill et al. [84] conducted a study using a modified polypyridyl ligand (Fig. 13), specifically 4,4′-Me_2_dppi (3,6-di-(4-methylpyrid-2-yl)pyridazine), to examine the Ru oxo redox reaction. This reaction resulted in the formation of two geometric isomers, both of which could be electrochemically oxidized to produce Ru(IV)-oxo. However, the outer isomer was found to be more reactive due to the instability of the in-isomers. Several benzyl alcohol derivatives, such as 1-phenyl ethanol and 1-phenyl-1-propanol, were examined as substrates in bulk electrolysis. It was shown that the catalytic rate decreased linearly with the increasing steric bulkiness of the alcohol. Furthermore, a study has documented the existence of Ru complexes [Ru(bpea)(bpy)(H_2_O)]^2+^ containing a tridentate poly pyridyl ligand called bpea (bpea = (N,N-bis(2-pyridyl)ethylamine))) [85].

**Fig. 14:**
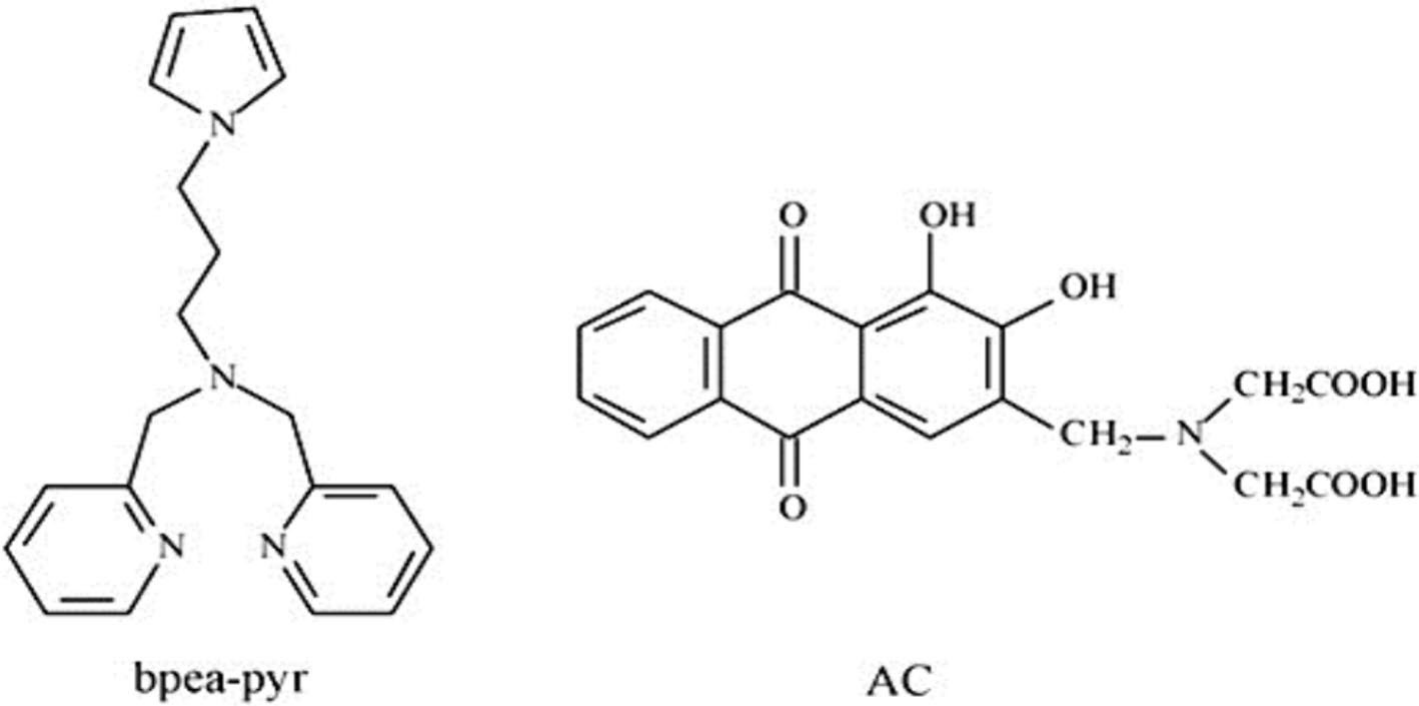
Structure of bpea-pyr and AC ligands [86, 87]

Recently, poly-[Ru(bpea-pyr) (bpy-pyr) (H_2_O)]^2^ + (Fig. 14) (bpea-pyr = bis-pyridin-2-ylmethyl-(3-pyrrol-l-propyl) amine, bpy-pyr = 4- methyl-4′-(4-pyrrol-1-yl-butyl)-[2,2′]-bipyridine) has been reported in the form of polymer film on the electrode and used towards oxidation of benzyl alcohol electrochemically. The results indicated that the redox cycle of immobilized Ru catalyst increases remarkably upto 922 cycles. Another similar work is [Ru(tpy)(*bpy)(OH)_2_]^2+^ (*bpy = 4′-methyl-(2,2′- bipyridine)-4-acetic acid) applicable as adsorbed catalyst on graphite chloride [86, 87].

**Fig. 15:**
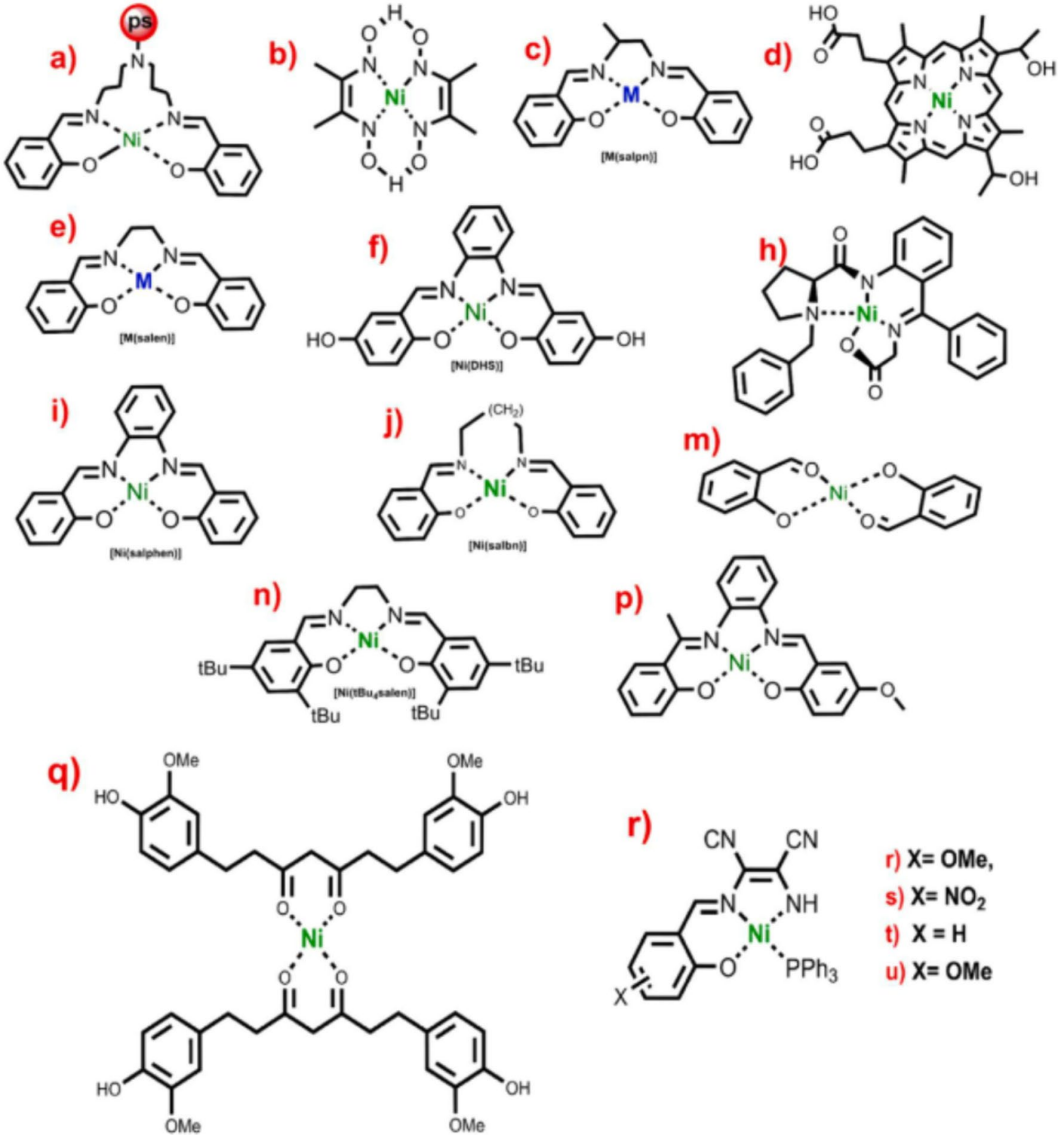
Chemical structure of a few metal complexes, which were used to catalyze the alcohol oxidation reaction [88]

In majority cases, the catalyst may undergo grafting on graphite felt leading to enhancement of rate of electrooxidation of alcohol. Other than Ru, and In-Sn oxide electrode modified with high valent trans-di-oxorhenium catalyst was demonstrated by Tanaka et al. [88]. The results of the study suggested that catalytic oxidation of 1-phenyl ethanol feel hardship in CH_2_Cl_2_ with rhenium (V) complex as catalyst, however, electrocatalytic oxidation could achieved higher catalyst turnover number as well as high current efficiency via immobilization of the rhenium(V) complex onto electrode surface. Figure 15 shows the chemical structure of MOR significance coordination compounds.

**Fig. 16:**
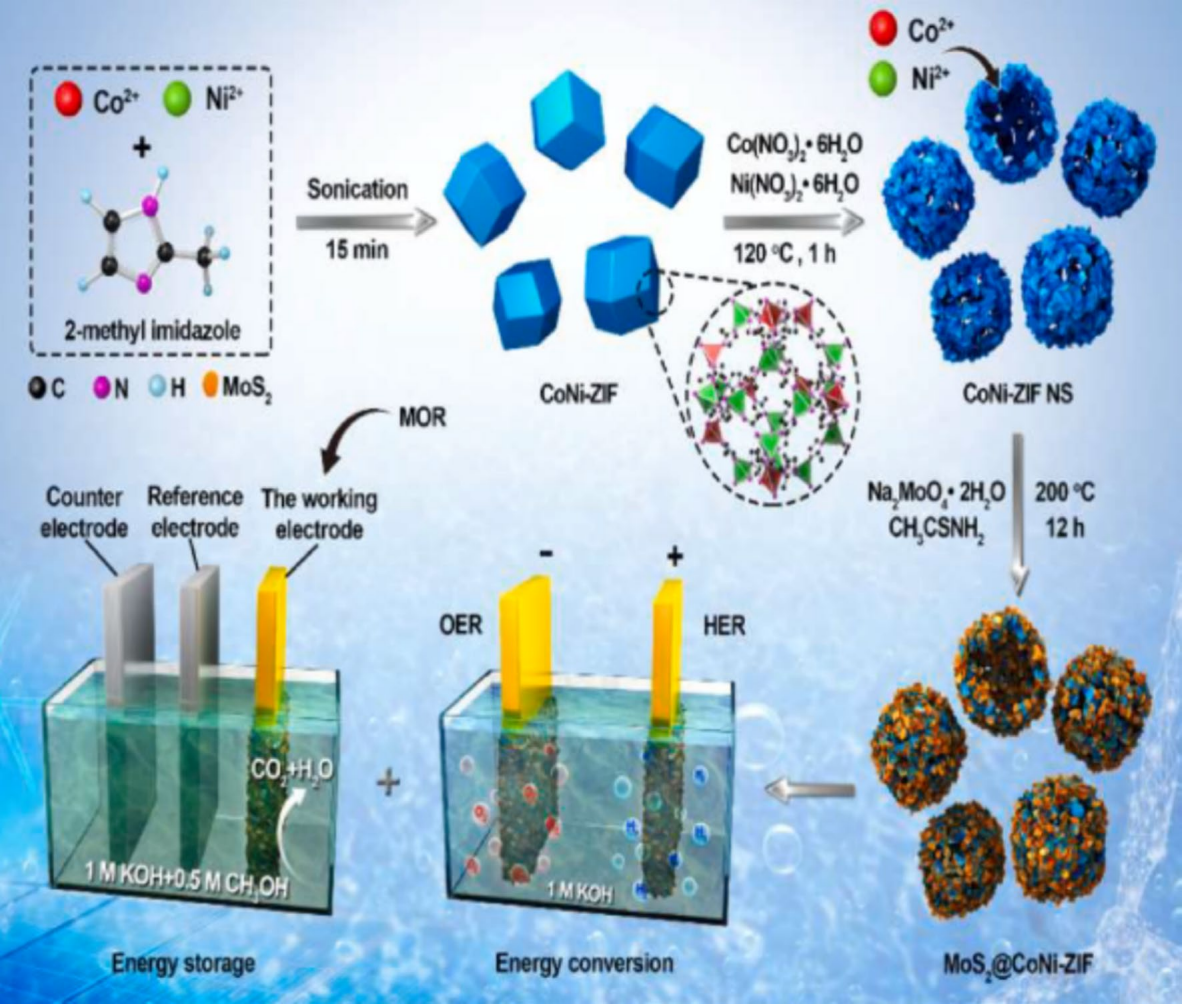
Synthesis protocol, and MOR, HER/OER applications of the CoNi-ZIF material. Adapted with permission [93]. Copyrights © 2019, Elsevier

While these molecular models aid in the oxidation of various alcohols like methanol, ethanol, and benzyl alcohol, they can also produce additional molecular models for the MOR process [89–91]. Alternatively, the combination of redox-active molecule ligands with transition metals can create an additional class of crystalline materials known as monometallic and bimetallic molecular organic frameworks (MOFs) [92]. These materials have the potential to serve as effective electrocatalysts for the MOR process. An additional benefit of using bimetallic MOFs with cobalt (Co) and nickel (Ni) could be helpful in the methanol electro-oxidation process. Liu and his colleagues created the CoNi-ZIF electrode material, which is a NiCo-ZIF@-MoS_2_ mix, as a change to the GCE (Fig. 16) [93]. When methanol was not present, the electrode exhibited a redox couple at 0.42 V and 0.26 V. The Ni^2+^/Ni^3+^ and Co^2+^/Co^3+^ redox couples, respectively, facilitated the oxidation and reduction reactions. Furthermore, the proportion of metal ions had a contingent influence on the electrochemical reaction of the bimetallic MOF. The bimetallic CoNi-ZIF (3:1) exhibited the highest current density when compared to other ratios. In addition, the electrode exhibited a proportional reaction to various methanol concentrations ranging from 0.2 to 0.7 M and achieved a peak current density of 72 mA. With an applied potential of 1.55 V, the composite demonstrated its ability to actively facilitate the process of water splitting, resulting in a current density of 10 mA.

**Fig. 17:**
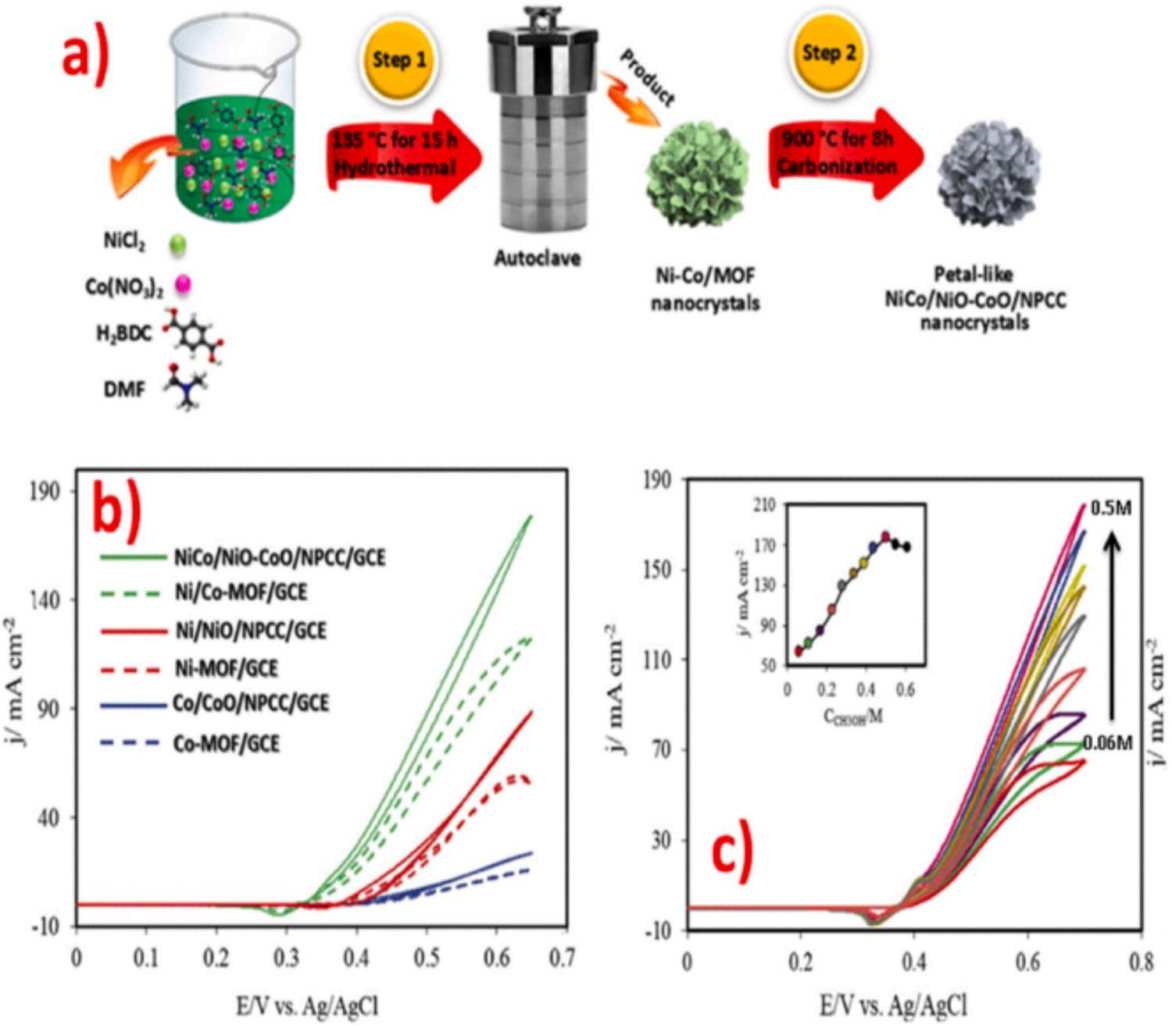
**a** Preparation strategy, CVs, **b** in absence of MeOH, **c** in presence of MeOH, for NiCo/NiO-CoO/NPCC composites. Adapted with permission [94]. Copyrights © 2019, Elsevier

A subsequent investigation conducted by Rezaee et al. [94] provided a comprehensive analysis of the alteration of a GCE (Glassy Carbon Electrode) for the electro-oxidation of methanol. This was achieved by utilizing a NiCo-BDC MOF (Metal–Organic Framework) as a sacrificial substance, resulting in the production of a composite material consisting of NiCo/NiO-CoO/nanoporous carbon (as depicted in Fig. 17a). The nanocomposite was made by directly pyrolyzing the bimetallic NiCo-MOF, as depicted in Fig. 11a. When MeOH was present, the composite exhibited a maximum anodic current of 185 mA when measured in alkaline conditions using CV (Fig. 17b, c). The improved catalytic efficiency arises from the formation of metal oxides and the conductive nature of the porous carbon structure. The proposed mechanism for the methanol electro-oxidation process (MOR) entails the direct formation of highly oxidizing MOOH molecules from metal oxides [94].

## Conclusions

To summarize, this review examined the recent progress made in electrocatalysts based on both noble and non-noble metals for the methanol oxidation reaction (MOR), which is a crucial step in direct methanol fuel cells (DMFCs). Moreover, this study underscores the importance of comprehensively understanding the mechanisms of methanol oxidation reactions (MOR) in different types of electrolytes, along with the essential factors that govern electrocatalysts. The research findings demonstrated that Pt-based materials supported on heteroatom-doped carbon matrix exhibited higher activity and stability towards MOR. Furthermore, by anchoring the metallic nanoparticles to nanocarbon supports, it is possible to get a consistent distribution of metallic sites that form strong bonds with heteroatoms (such as N, S, and P), leading to exceptional MOR performance. Since Pt-group metal-based catalysts are expensive in nature, it remains remains a major obstacle despite the effectiveness of this approach for MOR. Besides, the combination of Pt-group metals with affordable metals such as Mn, Fe, Co, Ni, Cu, etc. also exhibited remarkable MOR activity. This method can decrease the amount of noble metal used in the catalyst ink and decrease the overall cost of the catalyst, which is intriguing. On the other hand, atomically precised single-atom and dual-atom catalysts also displayed promise towards MOR. However, it is highly challenging to create catalysts that possess precise spatial arrangements for single or dual atomic sites. These catalysts often offer excellent activity as well as durability towards MOR. Moreover, due to the similarity of these catalysts with molecular systems, they offer a good understanding of catalytic reaction mechanisms at the atomic level.

Although there has been notable advancement in MOR electrocatalysts, there are still practical challenges that need to be addressed. To advance Pt-based catalysts, it is crucial to comprehend the MOR mechanism occurring at the level of individual metal atoms or nanoparticles. The sizes of metal electrocatalysts supported on a carbon substrate might vary within specific boundaries, encompassing individual atoms, nanoparticles, or clusters. Improving the loading and durability of single or dual atomic catalysts is essential for enhancing their performance in direct methanol fuel cells (DMFCs). Some molecular models have been proposed for alcohol oxidation reactions; however, they do not match the practical requirements for MOR due to their susceptibility to deterioration in the electrolyte.

In order to optimize the performance of both noble and non-noble metal electrocatalysts, several future directions should be considered. Firstly, it is important to develop environmentally friendly synthesis protocols for creating innovative electrocatalysts for MOR. Secondly, a variety of catalytic materials, consisting of both single and dual metallic catalysts, should be designed, and manufactured to establish structural and geometrical correlations. Thirdly, systematic in-situ and ex-situ investigations should be conducted to observe the inherent electrical, structural, and surface characteristics. Lastly, based on the findings from these characterizations, it is crucial to develop structural-reactivity descriptors to gain a deeper understanding of the mechanisms involved in MOR.

## Data Availability

No datasets were generated or analysed during the current study.
